# Risky Behaviours of Spanish University Students: Association with Mediterranean Diet, Sexual Attitude and Chronotype

**DOI:** 10.3390/nu13114042

**Published:** 2021-11-12

**Authors:** Pedro Manuel Rodríguez-Muñoz, Juan Manuel Carmona-Torres, Cristina Rivera-Picón, Ignacio Morales-Cané, Fabio Fabbian, Roberto Manfredini, María Aurora Rodríguez-Borrego, Pablo Jesús López-Soto

**Affiliations:** 1 Department of Nursing, Instituto Maimónides de Investigación Biomédica de Córdoba, 14005 Córdoba, Spain; juanmanuel.carmona@uclm.es (J.M.C.-T.); n82mocai@uco.es (I.M.-C.); en1robom@uco.es (M.A.R.-B.); n82losop@uco.es (P.J.L.-S.); 2 Department of Nursing and Physiotherapy, Universidad de Salamanca, 37007 Salamanca, Spain; 3 Faculty of Physiotherapy and Nursing, Universidad de Castilla-La Mancha, 45071 Toledo, Spain; 4 Faculty of Health Sciences, Universidad Pontificia de Salamanca, 37002 Salamanca, Spain; criverapi@upsa.es; 5 Department of Nursing, Universidad de Córdoba, 14004 Córdoba, Spain; 6 Department of Nursing, Hospital Universitario Reina Sofía de Córdoba, 14004 Córdoba, Spain; 7 Faculty of Medicine, Surgery and Prevention, University of Ferrara, 44121 Ferrara, Italy; f.fabbian@ospfe.it (F.F.); mfr@unife.it (R.M.)

**Keywords:** Mediterranean diet, healthy habits, alcohol consumption, university students

## Abstract

The most common drugs that are consumed by young people are alcohol and tobacco, which are especially prevalent in universities. These risk behaviours can be determined by a series of intrinsic and extrinsic factors. The aim of this study was to evaluate the consumption of alcohol and tobacco by Spanish university students and the relationship between the Mediterranean diet, sexual attitudes and opinions, and chronotype. A multicentre observational study enrolled 457 students from two public universities in Spain. The study period was from December 2017 to January 2018. The majority of the participants consumed alcohol (90.2%), tobacco consumption was low (27.2%), with a high percentage of students (78.6%) having a low dependence on nicotine. The surveyed students demonstrated a high adherence to the Mediterranean diet, which was shown to be associated with less risky alcohol consumption. The Mediterranean diet is a part of healthy lifestyle, and avoiding heavy drinking results in the intention to maintain such a lifestyle. In addition, unhealthy eating habits (skipping breakfast, eating sweets and pastries daily, and fast-food consumption) had a tendency to induce risky alcohol consumption. Therefore, to promote healthy lifestyle habits, it is considered important to establish programs that promote healthy diets in university settings and to evaluate them periodically.

## 1. Introduction

Drugs are psychoactive substances that affect perception, mood, awareness, and behaviour [1]. In 2019/2020, the most consumed drugs in Spain were alcohol (77%), followed by tobacco (39.4%) [2].

The initiation and increased consumption of these two substances occur above all in youth and frequently coincides with the university period [2]. The beginning of consumption occurs in response to society’s requirements and is influenced by factors such as not fitting in with one’s social group or not liking others, feeling more secure and confident, and being more sociable [3].

In respect to nutrition, university students are considered a vulnerable group [4]. During the university period, most students are living alone for the first time, meaning that they also become responsible for their own diet for the first time. They often cut down or skip meals, prefer to eat fast food, snack between meals, and consume alcohol regularly [4]. University students typically follow a diet that is high in fat and low in dietary fibre [4], with low consumption of fruits and vegetables, which is contrary to the principles of a Mediterranean diet (MD) [5]. The university stage is important for the development of eating habits and future health [4]. These habits are determined by an individual’s knowledge of nutrition and food—the more information someone has, the healthier their eating habits are. However, social, cultural, and economic factors as well as food preferences can influence diet [4,6]. In Spain, the majority of people follow a Mediterranean diet, which is a healthy diet [7]. Previous research has revealed that the low adherence of Spanish university students to a Mediterranean diet is related to high alcohol consumption [5,8], smoking, low levels of physical activity (PA), living alone, and leading a sedentary lifestyle [5], and good adherence to the Mediterranean diet is associated with an improved quality of life related to perceived health [9] and low body weight [5].

In the sexual sphere, alcohol consumption is associated with unsafe and undeclared sexual practices that are associated with condom use, giving rise to a higher risk of contracting sexually transmitted diseases or unwanted pregnancies [10]. Risky sexual practices in young people are a public health concern [11]. College students are more likely to engage in risky sexual behaviour and higher alcohol consumption than non-college youth [12,13].

Attitudes towards sexuality and sexual opinions can be measured by the erotophobia–erotophilia dimension, which dispositions that are followed to respond to sexual stimuli. Erotophobia is the response that indicates more negative attitudes to a sexual stimulus and feelings of avoidance, and erotophilia is a behaviour that contrasts with erotophobia, demonstrating a positive attitude towards a sexual stimulus and the desire to look for such a stimulus [14,15].

The majority of Spanish youth have their first sexual encounter between the ages of 16 and 20 years old [16]. Many young people use substances, specifically alcohol but also cannabis, cocaine, and ecstasy, for the effects that they can have to facilitate sex, from relaxation to disinhibition [17]. The abuse of these drugs is related to risky sex, which contributes to sexually transmitted diseases more than safe sex [18].

Drugs are used more in areas with active nightlife and during weekend contexts and are related to the possibility of starting a sexual encounter [19]. In addition, university students also have a more evening-centric personal circadian tendency (chronotype), which, in turn, is associated with risky behaviours [20].

For all of these reasons and given the relationship between tobacco and alcohol consumption, dietary habits, sexual opinions and risky sexual practices, our study aimed to evaluate the consumption of alcohol and tobacco by Spanish university students and the relationship between the Mediterranean diet, sexual attitudes and opinions, and chronotype.

## 2. Materials and Methods

### 2.1. Study Design and Participants

A multicentre observational descriptive study was conducted. The study subjects were university students from the Universities of Cordoba (UCO) and Castilla La Mancha (UCLM) in Spain. The study was designed to cover all students from both universities. Inclusion criteria: Students enrolled at the UCO and UCLM in Undergraduate, Master, and Doctorate studies.

### 2.2. Data Collection

The data were collected from 8 December 2017 to 31 January 2018. An online questionnaire was prepared using the Google Forms system (Google Inc., Mountain View, CA, USA), which is a free tool and uses a user-friendly web interface, that ensured the confidentiality of the users and included the Information Sheet and Informed Consent. The link to the questionnaire was sent to those responsible for the dissemination of the study by email.

### 2.3. Instruments

In the survey, the six questionnaires described below were used:

Sociodemographic data were collected in a self-prepared questionnaire that included questions pertaining to sex, age, university and university degree, place of residence, marital status, academic year, and variables related to the participants’ parents healthy and unhealthy habits.

For alcohol consumption, the Alcohol Use Disorders Identification Test (AUDIT) [21] (Spanish version [22]) was used. The AUDIT test is a questionnaire that is made up of 10 items, whose scores range from 0 to 40 points. Each item has five response options, with a score between 0 and 4, with the exception of items 9 and 10, which only have three possibilities with scores 0, 2, 4. The first three questions refer to alcohol risk consumption, questions 4 to 6 measure dependency symptoms, and the last four questions measure harmful alcohol consumption [23]. For university students [24], scores between 8 and 12 for men and 6 and 12 for women refer to risky consumption. Scores from 13 points for both sexes denote alcohol dependence. Binge drinking was measured with the third question from the AUDIT test. Responses were categorized as: never, less than once a month, and monthly or more (including monthly, weekly, and daily or almost daily) [11,25].

The Fagerstrom Nicotine Dependence Test was used to measure nicotine dependence [26] (Spanish version [27]). This questionnaire contains a scale comprising six items. This scale has cut-off points at 4 and 7. Less than 4 points denotes low dependence, a score between 4 and 7 denotes a moderate dependence, and a score of more than 7 denotes a high dependence.

The Richmond Test [28] was used to measure motivation to quit smoking (Spanish version [29]). It is a questionnaire that consists of four items assessing the degree of motivation to quit smoking. The test scores range from 0 to 10 points. The level of motivation and the cessation of tobacco use scores are null or low (from 0 to 3), doubtful (between 4 and 5), moderate and in need of help (from 6 to 7), and high (from 8 to 10).

The Mediterranean diet quality index for children and adolescents (KIDMED) [30] consists of 16 questions with answers comprising two options (yes/no). Twelve questions are related to aspects consistent with the Mediterranean diet, and four discuss to unrelated aspects. The scores range from 0 to 12 points. Scores of up to 3 points are considered as a low adherence to the Mediterranean diet; scores from 4 to 7 points denote a diet with medium adherence; and scores from 8 to 12 denote a high adherence to the Mediterranean diet. Scores are obtained as follows: if you answer affirmatively to questions related to diet, 1 point is added, if you answer affirmatively to questions that are related negatively to diet, 1 point is subtracted.

The Revised Sexual Opinion Survey (R-SOS) [14] was used to collect sexual opinions.

This scale is made of 20 questions with a likert response scale that ranges from 1 to 7. A score of 1 would denote totally disagree, and a score 7 would denote totally agree. This questionnaire measures the erotophobia–erotophilia dimension, which is defined as the willingness to respond to sexual stimuli and ranges from a negative point, erotophobia, to a positive point, erotophilia. A negative response to sexual stimuli would be erotophobia, and a positive response would be erotophilia.

For chronotype, we used the reduced scale of Horne and Östberg’s morningness–eveningness questionnaire (rMEQ) [31]. This scale is validated and was adapted to the Spanish student population [32]. The questionnaire consists of five items, with five answers for questions 1, 3, and 4, and questions 2 and 5, only have answer options. The score ranges from 4 to 25 points, with higher scores being associated with morningness, meaning that the respondent has morning preferences, and lower scores being eveningness, denoting that the respondent has an evening chronotype (E-type), with evening preferences.

### 2.4. Statistical Analysis

Data analysis was performed using the statistical program International Business Machines (IBM) Statistics Package for the Social Sciences (SPSS) version 23 (IBM Corp, Armonk, NY, USA). Continuous variables were stablished through mean and standard deviation (SD). Percentages (%) were used for categorical variables. A comparison of the proportions of the categorical variables was also performed using the chi-square or Fisher’s tests for the contingency tables. Analysis of variance (ANOVA) was also performed to analyse the differences between two or more means. Pearson’s correlation analysis was conducted to evaluate a possible linear correlation. Adjusted linear regression analyses were conducted when any significant association existed. In all statistical tests, testing was significant when *p* < 0.05.

### 2.5. Ethical Considerations

The study was admitted by public institutions associated with the study. Additionally, it received a positive report from the Ethics Committee.

## 3. Results

### 3.1. Participants Characteristics, Alcohol Consumption, Tobacco Consumption, and Sexual Opinion

In total, 464 students participated in the study, and 7 were excluded due to incomplete questionnaires. The final sample consisted of 457 students. Of the respondents, 33.3% were men, and 66.7% were women. The mean age was 20.93 ± 3.28. A total of 36.5% of the participants were UCO students, and 63.5% were UCLM students.

The majority of the participants (90.2%) consumed alcohol. The men showed a greater alcohol risk tendency than the women did (*p* = 0.027) (Table 1). Students whose parents consumed alcohol also had a greater tendency for risky alcohol consumption (*p* = 0.002). Although there was no statistical significance, students whose parents practiced physical activity were at a higher risk of alcohol consumption (*p* = 0.059).

A total of 51.4% of students consumed alcohol while engaging in binge drinking. About 34.1% binge drank less than once per month, and 17.3% of participants did so monthly or more frequently.

A total of 27.6% of the university students consumed tobacco. Among the students who consumed tobacco, 78.6% had a low dependence on nicotine, 19.8% had a medium dependence, and 1.6% a high dependence.

With respect to marital status, significant differences were observed (*p* < 0.01), with 83.1% of singles having low dependence, while only 14.3% of married people were in this category. Approximately 94.4% of students whose parents had a history of alcohol consumption had a low nicotine dependence (*p* < 0.01).

Half of university students (49.2%) were motivated to quit smoking, of which 15.9% had high motivation. Concerning the branch of studies, the students with the highest motivation to quit smoking were Health Sciences students (28%).

The mean score for the sexual opinions of the university students was 77.93 ± 19.92. Men showed a higher mean of erotophilia (82.66 ± 18.30) than women (75.46 ± 20.32), and the UCO students scored (78.11 ± 19.06) while those at UCLM had a score of (77.82 ± 20.46).

Students who used protection had a higher mean erotophilia score (78 ± 19.69) than those who did not (77.15 ± 22.66).

### 3.2. Correlation between Responses for Each Item of the KIDMED Score and AUDIT Score

Table 2 shows that the subjects who declared eating fruit or drinking fruit juice every day (*r* = −0.116, *p* < 0.05), liking pulses and eating fruit more than once a week (*r* = −0.100, *p* < 0.05), using olive oil at home (*r* = −0.163, *p* < 0.01), and eating two yogurts and/or some cheese (40 g) daily (*r* = −0.118, *p* < 0.05) had a lower tendency to drink heavily.

On the contrary, subjects who declared going to a fast-food restaurant more than once a week (*r* = 0.169, *p* < 0.01), skipping breakfast (*r* = 0.096, *p* ≤ 0.05), and eating sweets and candy several times every day (*r* = 0.115, *p* < 0.05) had a higher tendency to engage in risky alcohol consumption.

### 3.3. Motivation to Quit Smoking Is Associated with Less Tendency to Erotophilia, E-Type Chronotype, and Adherence to MD

Students with high values in motivation to quit smoking showed lower erotophilia tendencies and low chronotype values (E-type), being evening oriented. Although no significant differences were found, those who had high values in adherence to the Mediterranean diet tended to have greater motivation to quit smoking (Table 3).

### 3.4. Risky Alcohol Consumption Associated with Adherence to MD and Nicotine Dependence

Students with high scores in risk alcohol consumption showed less adherence to the Mediterranean diet (β= −0.439, *p*= 0.006) Those who were highly dependent on nicotine tended to engage in less risky alcohol consumption (Table 4).

## 4. Discussion

In this study, university students with a high adherence to the MD showed lower scores for risky alcohol consumption. The few studies that were found that associate the Mediterranean diet with risky alcohol consumption have not detected a relationship between both variables [33], although various authors have stated that a risky (unhealthy) pattern of alcohol consumption is accompanied with unhealthy eating habits in addition to less adherence to dietary guidelines [34,35,36]. Other studies identify alcohol consumption as a barrier to motivation among university students to eat a healthy diet [37].

On the other hand, the Mediterranean diet, although it is not part of an item in the questionnaire used in the study, includes moderate alcohol consumption, the beverages being beer and red wine [38].

In addition, the eating behaviours surrounding alcohol consumption in college students are influenced by friendships, socialization, availability, and cost, with this consumption of alcohol and food occurring in an environment with negative health behaviours [39].

The items related to the unhealthy aspects of the questionnaire determining adherence to the Mediterranean diet are correlated with a higher score in the AUDIT score, indicating higher risky alcohol consumption. According to other studies, having good breakfast habits [40] and having breakfast daily [41] are positively associated with lower consumption of alcohol and tobacco. “Taking sweets and candy several times every day” and its relationship with higher risk alcohol consumption has been shown in other studies [42,43]. Specifically, in Spain, coinciding with the confinement by COVID-19 pandemic, there was an increase in the purchase of alcoholic beverages along with sweets and pastries [42]. This is an example of a circumstance that demonstrates how the environment determines the type of behaviour. In the same line, fast food consumption is also correlated with higher risk alcohol consumption, as demonstrated by other studies in university students [43].

Although there is no statistical significance, students whose parents practice physical activity had a higher risk alcohol consumption. No studies have been found a link the parenting practicing PA with the consumption of alcohol, but there are studies that mention that PA offers benefits for the prevention, reduction, and treatment of alcohol and the consumption of other substances [44]. On the other hand, in a study of Austrian university students, it seems that between PA and alcohol consumption, there is no significant relationship to reduce this consumption tendency [45].

In terms of gender, studies show men engage in higher risk alcohol consumption and more alcohol consumption [46,47,48,49,50], suggesting that being a man is a risk factor for alcohol consumption [16,45,51]. Despite the differences in alcohol consumption by gender [52], consumption is increasing in women, with progressively less difference being present within men [53]. It is necessary to give some importance to equal consumption between men and women due to the gender differences in alcohol metabolism, with more harmful effects being found in women.

In turn, more than three quarters of the studied population, 78.6%, had a low dependence to nicotine. This low nicotine dependence may be due to starting to smoke at the university when the number of smokers increases, as other studies point out [13]. The data compel the proposal of health promotion interventions for university students in this area to achieve reductions in tobacco consumption, as students do not have high nicotine dependence.

Half of the study population was motivated to quit smoking, whether high, moderate or doubtful, and the other half had low or no motivation. These motivational data reinforce the proposal to implement interventions for university students related to the promotion of health materials related to consumption and/or addictions. The proposed activities could include protective factors against tobacco consumption and nicotine dependence in family settings. As other authors have indicated [54], purchase, sale, and consumption can be regulated in the university environment and surroundings, which can be reinforced with information on the health problems related to smoking [55].

No studies have been found that link nicotine dependence with risky alcohol consumption, but there are authors who link nicotine with an increase in alcohol-related behaviours [56], increasing the desire to consume alcohol [57].

Differentiating by the branch of study, Health Sciences students have the highest motivation to quit smoking (28%). These data may be due to the acquired knowledge about healthy habits. Others [55] point out that the main reasons to quit smoking are health aspects, ranking above personal relationships, financial resources, and environmental impact.

Another aspect to highlight in the present study is that it has been observed that smokers who are motivated to quit smoking have a lower tendency for erotophilia, the E-type chronotype, and adherence to the Mediterranean diet. Gibson et al. [58] reported how smoking affects the circadian rhythm, producing insomnia, which reduces the chances of quitting smoking. The authors also observed how smoking more cigarettes decreases the probability of having a morning chronotype. Other studies also corroborate these data, associating smoking with a later chronotype (E-type) [59]. In relation to adherence to a Mediterranean diet, several studies show that having good breakfast habits and eating breakfast daily is associated with lower tobacco consumption [40,41].

The mean score for sexual behaviour is 77.93, ranging between 0 to 120 (SD 19.927). Men showed the highest degree of erotophilia, as confirmed by other studies [60,61] although the difference in scores by gender was small [61].

Students who declare safe sex behaviours reported a tendency for erotophilia compared with those who practiced risky sex. Similar data were found in other studies [62], which associated erotophilia with greater accessibility to condom use. These data also connect erotophobia with the non-use of sexual protection, possibly due to not having such active sexual behaviour and the same access and availability to condoms as their counterparts with erotophilic behaviour [62,63].

This investigation has some limitations. Firstly, it is a cross-sectional study (coinciding with non-school period), so the present results could not determine the temporal direction of associations. Secondly, due to the non-response rate and the possible difficulty of completing the questionnaire, it is unknown whether our findings are representative of all Spanish university students. For future work, more direct physiological measures or use of diaries/journals should be considered to measure behaviour, and the social class of the students should be considered. Nonetheless, the results of the study conducted here were collected at two universities in two different areas of Spain and were consistent with the existing literature.

## 5. Conclusions

Although daily low or moderate wine consumption is part of the Mediterranean diet, higher alcohol consumption (risky alcohol use) is against its principles. In this study, the high adherence of Spanish university students to the Mediterranean diet was associated with less risky alcohol consumption. In addition, the unhealthy eating habits of the respondents (skipping breakfast, eating sweets and pastries daily, and fast-food consumption) were related to their higher risky alcohol consumption. The relationship of adherence to the Mediterranean diet and avoiding heavy drinking may result from the desire to lead a healthy lifestyle. Motivation to quit smoking is associated with a lower tendency for erotophilia and the eveningness chronotype. It can be seen how healthy lifestyle habits such as eating a healthy diet can promote lower alcohol consumption. These data become more important when produced in a university environment since the habits acquired at the university stage can determine later habits that, in turn, determine the future lifestyle of each person. For this reason, in order to promote healthy lifestyle habits, it would be important to conduct interventions and programs that promote healthy diets in university settings and to evaluate these programs periodically.

## Figures and Tables

**Table 1:** Sociodemographic variables and AUDIT Score.

Sociodemographic Variables	AUDIT Score		
	*n* (Mean)	SD	*p*
Gender			0.027
Male	140 (6.02)	4.42	
Female	275 (5.04)	4.14	
University			0.001
UCO	150 (4.54)	3.31	
UCLM	265 (5.84)	4.64	
History of alcohol consumption by parents			0.002
Yes	114 (6.41)	4.74	
No	301 (4.98)	3.99	
History of tobacco consumption by parents			0.244
Yes	271 (5.55)	4.44	
No	144 (5.04)	3.88	
History of physical activity by parents			0.059
Yes	29 (7.9)	7.35	
No	386 (5.18)	3.88	
Use of a protection method when having sexual relations			0.092
Yes	382 (5.19)	3.85	
No	33 (7.42)	7.28	
Place of residence			0.172
University residence	36 (5.5)	4.75	
Cohabit with parents	186 (4.87)	3.45	
Cohabit with peers	181 (5.93)	4.88	
Live with a partner	2 (4)	2.07	
Live alone	3 (7.66)	4.04	
Marital status			0.546
Single	399 (5.28)	4.08	
Married	15 (5.93)	3.26	
Types of studies			0.583
Health Sciences	125 (5.74)	4.52	
Social and Legal Sciences	94 (5.51)	4.52	
Engineering and Architectures	27 (4.37)	2.60	
Arts and Humanities	77 (5.16)	4.36	
Physical and Life sciences	92 (5.2)	3.90	

*n*, count; SD, standard deviation; UCO, University of Cordoba; UCLM, University of Castilla-La Mancha; AUDIT score: 6–12 points for women and 8–12 points for men (risk consumption); from 13 points (alcohol dependence).

**Table 2:** Correlation between AUDIT score and responses for each item of the KIDMED score.

KIDMED Test (%, Yes)	AUDIT Score	
	Pearson’s Correlation (r)	Sig. (Bilateral)
Ingests fruit or fruit juice every day	−0.116	0.019
Has a second serving of fruit every day	−0.044	0.371
Has fresh or cooked vegetables regularly once a day	−0.043	0.380
Has fresh or cooked vegetables more than once a day	−0.043	0.038
Consumes fish regularly (at least 2–3 days/week)	−0.081	0.099
Goes to a fast-food (hamburger) restaurant more than once a week	0.169	0.001
Likes pulses and eats them more than once a week	−0.100	0.042
Consumes pasta or rice almost every day (≥5 times/week)	0.037	0.451
Has cereals or cereal products (bread) for breakfast	−0.018	0.710
Consumes nuts regularly (at least 2–3 times per week)	0.074	0.134
Uses olive oil at home	−0.163	0.001
Skips breakfast	0.096	0.050
Has a dairy product for breakfast (yogurt, milk, etc.)	−0.037	0.447
Eats two yogurts and/or some cheese (40 g) daily	−0.118	0.016
Has commercially baked goods or pastries for breakfast	0.078	0.115
Takes sweets and candy several times every day	0.115	0.019

**Table 3:** Associations between adherence to the Mediterranean diet, chronotype, erotophobia–erotophilia dimension, and motivation to quit smoking.

	Motivation to Quit Smoking		
	β	95% CI	*p*
Adherence to the Mediterranean diet	0.142	−0.041, 0.325	0.127
Chronotype	−0.186	−0.322, −0.050	0.008
Erotophobia–erotophilia dimension	−0.032	−0.053, −0.011	0.003

CI, confidence interval. Linear regression models were used to establish associations with continuous variables (adherence to the Mediterranean diet, chronotype, erotophobia–erotophilia dimension, and motivation to quit smoking). R^2^ Nagelkerke: 0.095.

**Table 4:** Associations between adherence to the Mediterranean diet, nicotine dependence, and risk alcohol consumption.

	Risk Alcohol Consumption		
	β	95% CI	*p*
Adherence to the Mediterranean diet	−0.439	−0.790, −0.089	0.006
Nicotine dependence	−0.602	−0.172, 1.031	0.008

CI, confidence interval. Linear regression models were used to establish associations with continuous variables (adherence to the Mediterranean diet, nicotine dependence, and risk alcohol consumption). R^2^ Nagelkerke: 0.149.
